# A diamond opportunity

**DOI:** 10.1039/d0sc90281k

**Published:** 2020-12-24

## Abstract

A welcome to 2021 from May Copsey, Executive Editor, *Chemical Science*.
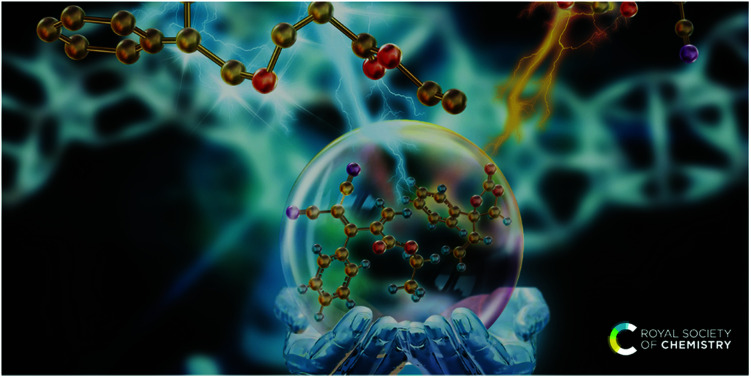

Diamond or platinum open access; open and free, for authors and readers. However you describe it, being open and accessible is at the heart of what we are aiming to achieve in *Chemical Science*.

## New Perspectives

It's not just about who can read our articles, we also want to ensure we are open in many other ways too, such as being receptive to ideas that challenge the *status quo*. We want articles in *Chemical Science* to provoke thought and to inspire new directions and perspectives. This is why we have been giving the purpose of the reviews in the journal some thought.

Ten years ago, when we launched *Chemical Science*, we offered the option to authors of publishing Mini-Reviews or Perspective articles. Now is a good time to reflect on this, and we realised that we’d like to refresh these article types to emphasize how the journal is developing. This will be two-fold:

(1) We are evolving our existing Mini-Reviews to be simply Reviews. This is to provide our authors with a more flexible review article type that will do away with length restrictions. This approach will complement the Edge article (which has been doing this for ten years for primary research).

(2) We are refocussing our Perspective articles. These will now be a more personalised account of a research area. We aim to provide a platform for authors to provide a personal or speculative viewpoint on a given subject and its future development. To encourage authors to think about how the field will develop over the next 5–10 years, we are including a ‘Future outlook’ section. We also want authors to comment on how they see their own research contributing to the future direction of an area. Contributions by early career researchers are particularly welcome.

Both article types will continue to be subject to the same level of rigorous peer-review as is used for our current reviews and Edge articles. Perspectives and Reviews are normally published by invitation of the *Chemical Science* Editorial Board. However, suggestions from authors are welcome and enquiries should be directed to the editorial office by email CHEMICALSCIENCE-RSC@rsc.org.

The formal description of these articles types is given below (Table 1) and is also available on our website. For examples do take a look at the ones that we have already published in issue 1 of 2021. Hopefully they offer some interesting food for thought on the importance of asymmetry in porous materials, by Alexandre Legrand, Zaoming Wang, Javier Troyano and Shuhei Furukawa (DOI: 10.1039/D0SC05008C), nanogenerators for self-charging power systems by Xiong Pu and Zhong Lin Wang (DOI: 10.1039/D0SC05145D), and the role of supramolecular approaches for artificial photosynthesis by Tom Keijer, Tessel Bouwens, Joeri Hessels and Joost Reek (DOI: 10.1039/D0SC03715J).

**Table 1:** Article type descriptions for Reviews and Perspectives published in *Chemical Science*

**Review article**
Reviews in *Chemical Science* must be authoritative, state-of-the-art accounts of the selected research field, focusing on the key developments that have shaped the topic, rather than comprehensive reviews of the literature. Reviews should be timely and add to the existing literature, rather than duplicate existing articles. Authors are encouraged to summarise important findings instead of re-iterating details already available in the primary work and should provide summary figures instead of multiple figures from original manuscripts, where appropriate.
The purpose of a Review is to bring the reader up to date with research in a particular field and to provide a critical assessment of recent developments. Since the readership of Chemical Science is broad, it is essential that the Review is easily comprehensible to a non-specialist in the field. Authors are encouraged to identify areas in the field where further developments are imminent or of urgent need. Please note that Reviews should not contain any original research.
Reviews can include photographs and brief biographies (max 100 words) for up to six authors, which must be supplied prior to acceptance. All Reviews will undergo a rigorous and full peer review procedure, in the same way as Edge primary research papers.
Written by leaders in their fields, Reviews are normally published by invitation of the *Chemical Science* Editorial Board. However, suggestions from authors are welcome and enquiries should be directed to the editorial office.

**Perspective**
A Perspective is a personalised account of a research area and provides a platform for authors to provide a personal or speculative viewpoint on a given subject and its future development. All Perspectives will undergo a rigorous and full peer review procedure, in the same way as regular research papers.
A Perspective should report a balanced account of the selected research field. If the author is focussing on their own research contribution to the field, then a balanced discussion of related work should be included to set the authors’ contribution within a wider context. Perspectives should be selective, focusing on the key developments that have shaped the topic, rather than comprehensive reviews of the literature. Authors are encouraged to summarise important findings instead of re-iterating details already available in the primary work and should provide summary figures instead of multiple figures from original manuscripts, where appropriate.
We encourage authors of Perspective articles to include personal biographies that will be placed at the start of the manuscript. This should give an overview of the author’s research career so far, their chosen area(s) of research and future goals. It is expected that this will be no more than 200 words in length and a photograph should also be included. A ‘Future outlook’ section should also be included at the end of the manuscript to provide a personal view of how the field will develop over the next 5-10 years and how the authors see their own research contributing to this.
Perspectives are normally published by invitation of the *Chemical Science* Editorial Board. However, suggestions from authors are welcome and enquiries should be directed to the editorial office. Contributions by early career researchers are particularly welcome.

## Credit where credit’s due

We want to make sure all of the authors featured in *Chemical Science* are given the credit they deserve. So we are now asking all our authors to now include an Author Contribution Statement in their future articles.

In 2020, the Royal Society of Chemistry signed the San Francisco Declaration of Research Assessment (DORA) and its principles highlight a need to assess research on its own merits.^1^ We have been encouraging the inclusion of Author Contribution Statements, however due to the importance of promoting responsible research assessment practices, we are taking the step of making this mandatory in future *Chemical Science* articles. We appreciate that this will take more time for authors to prepare, but we feel that recognition of those who have done all the hard work is truly important, and deserves to be called out in each article.

Guidelines on how to prepare these statements can be found on our website, under our author responsibilities.^2^ We’ll also be looking for ways we can further recognise the achievements of our authors and reviewers later this year.

## A reviewing challenge

It goes without saying that 2020 will be a memorable year for everyone, as it has thrown many challenges at us all. At the beginning of the year, we saw increasing submissions as researchers went into lockdown around the world. The pressure was on for our reviewers, who as we can all appreciate were also juggling family and caring responsibilities, as well as managing their groups remotely.

So I wanted to take this opportunity to particularly thank all our reviewers from this year. The primary purpose of the review process is to improve the quality of the research articles that are published, and so the work that *Chemical Science* reviewers do is crucial to ensuring readers can trust what they see in the journal.

However it is more than this, reviewer reports are also a way of training the next generation of researchers, as they take their first steps in the publication process. It is a way of sharing experience and knowledge, ideally in a positive and constructive way, which will help research projects and ideas to grow for the future. Perhaps now more than ever, it’s important for us to recognise the importance and value of that part of a reviewer’s role.

Finally on behalf of the whole *Chemical Science* team and Editorial Board, I’d like to take this chance to wish everyone a very happy New Year and a safe and happy 2021.

 

May Copsey

Executive Editor, *Chemical Science*

 

(1) https://www.rsc.org/news-events/articles/2020/jun/rsc-signs-dora

(2) https://www.rsc.org/journals-books-databases/journal-authors-reviewers/author-responsibilities/

## Supplementary Material

